# Development of national competence areas and competence goals for patient safety using a modified Delphi method

**DOI:** 10.1136/bmjoq-2025-003887

**Published:** 2026-03-31

**Authors:** Maria Unbeck, Anna Dahlgren, Christian Danielsson, Karin Pukk Harenstam, Helena Walfridsson, Mirjam Ekstedt, Axel Ros

**Affiliations:** 1 School of Health and Welfare, Dalarna University, Falun, Sweden; 2 Department of Clinical Sciences, Danderyd Hospital, Karolinska Institutet, Stockholm, Sweden; 3 Department of Clinical Neuroscience, Karolinska Institute, Stockholm, Sweden; 4 Department of Psychology, Stockholm University, Stockholm, Sweden; 5 National Board of Health and Welfare, Stockholm, Sweden; 6 Astrid Lindgren’s Children’s Hospital, Karolinska Universitetssjukhuset, Stockholm, Sweden; 7 Medical Management Centre, Department of Learning, Informatics, Management and Ethics (LIME), Karolinska Institutet, Stockholm, Sweden; 8 Faculty of Life and Health Sciences, Linnaeus University, Kalmar, Sweden; 9 Department of Learning Informatics Management and Ethics, Karolinska Institute, Stockholm, Sweden; 10 School of Health and Welfare, Jönköping Academy for Improvement of Health and Welfare, Jönköping University, Jönköping, Jonkoping County, Sweden; 11 Futurum – Academy for Health and Care, Region Jönköping County, Jönköping, Sweden

**Keywords:** Patient safety, Health professions education, Continuing education, continuing professional development

## Abstract

**Background:**

The WHO calls for integrating patient safety curricula in healthcare education globally, but the limited contextual applicability of existing frameworks constrains national implementation. This work aims to describe the development of Swedish national competence goals in patient safety to establish patient safety as a distinct field summarised within a comprehensive framework of competency areas.

**Method:**

The national competence goals were developed in a project initiated by the Swedish National Board of Health and Welfare that commissioned a group of academics with expertise in patient safety to work on the project. The development entailed an iterative process involving both physical and digital meetings, individual work and two rounds of questionnaires. Initially, drawing on expert knowledge and international literature, a set of competence areas based on key concepts in patient safety was proposed and defined. Within each competence area, several competence goals were developed. A modified Delphi process was then employed to collect insights from two multidisciplinary panels of experts (n=23). Finally, competence areas, key concepts and competence goals were refined based on feedback from the two Delphi panels.

**Results:**

The project resulted in a national framework comprising 15 competence areas and 113 competence goals, highlighting key dimensions of patient safety such as foundational concepts, professional roles, systems thinking, patient involvement, human factors, communication and teamwork, organisational culture, risk awareness, learning from adverse events, evaluation, safe practices, technology, leadership, emergency preparedness and high-risk care situations.

**Conclusions:**

The development of national competence areas and goals marks an advancement in establishing patient safety as a distinct scientific discipline, where they collectively provide a broad and structured set of educational goals and standards. This initiative provides a foundation for integrating patient safety curricula into national healthcare education and strengthening patient safety practices, which can serve as an inspiration to others.

**WHAT IS ALREADY KNOWN ON THIS TOPIC**

- The WHO urges all countries to integrate patient safety curricula into healthcare education, yet existing international frameworks often lack contextual relevance, limiting their effectiveness in local implementation.

**WHAT THIS STUDY ADDS**

- This study positions patient safety as a distinct scientific and educational discipline by developing a national framework consisting of 15 competence areas and 113 competence goals.
- The framework was developed using a modified Delphi method, ensuring expert feedback and methodological structure.

**HOW THIS STUDY MIGHT AFFECT RESEARCH, PRACTICE OR POLICY**

- The framework supports the systematic development and integration of patient safety education across all levels of healthcare, serving as a foundation for both policymaking and curriculum design.
- Research focusing on the development, implementation and effects of this initiative, as well as its impact on patient safety practices and outcomes, is necessary.

## Background

 The rapid development of healthcare, coupled with demographic shifts such as an ageing population and the increasing prevalence of chronic diseases, underscores the critical need for robust education programmes that acknowledge the increasing complexity in healthcare to maintain high standards of patient care and safety. To ensure sustainable and safe care, it is essential to strengthen and retain patient safety competence among healthcare staff.^1^

Health profession schools, including those for medicine, nursing, psychiatry, pharmacy and dentistry, often provide limited education on patient safety. Traditional curricula primarily focus on basic science and the acquisition of factual knowledge and clinical skills. However, they generally do not emphasise the essential concepts, attitudes and skills needed for safe practice and continuous improvement in care.^2^

The WHO has been at the forefront of developing educational frameworks to enhance patient safety. The WHO Global Patient Safety Action Plan 2021–2030^3^ urges all countries to integrate a patient safety curriculum into the education of healthcare professionals. The WHO Patient Safety Curriculum identified 11 essential topics to be included in patient safety education globally.^4^ This guide serves as a comprehensive resource for integrating patient safety principles into healthcare education. It aims to build capacity for patient safety education by providing ready-to-teach courses on crucial topics such as medication safety, infection control and effective communication. Similarly, the Canadian Patient Safety Institute developed a Safety Competencies Framework highlighting six patient safety competency areas that healthcare students should master, for example, being able to work in teams for patient safety, contribute to a culture of patient safety, manage safety risks and optimise human and environmental factors.^5^

Since these curricula were developed, there has been a trend in education to shift from root cause analysis, basic patient safety concepts and error reporting to include concepts such as communication, teamwork and human factors.^1^ In a systematic review of patient safety education interventions, the WHO curriculum was the most commonly used curriculum. However, there was a great variation in the number and types of topics included.^1^

### The Swedish context

Both the Swedish national action plan for increased patient safety^6^ and the audit conducted by the Swedish National Audit Office^7^ emphasise the crucial need for ongoing education and competence development to ensure patient safety and high-quality healthcare. The national action plan^6^ emphasises the importance of systematic patient safety work at all levels of healthcare, aiming to prevent harm and improve safety practices, including leadership, culture and competence development, to enhance patient safety. The Swedish National Audit Office^7^ highlighted the need to strengthen education and competence within the healthcare sector. It emphasised the importance of clear and coordinated governance to ensure the effective implementation of educational initiatives aimed at improving healthcare competence. In line with this, the National Board of Health and Welfare (NBHW) initiated an evaluation of the WHO curriculum from a Swedish perspective in 2021^8^; the WHO curriculum was seen as a valuable inspiration for a national curriculum. However, the evaluation identified the need to incorporate more recent knowledge from safety science and to develop a national Swedish curriculum also based on understanding of the Swedish healthcare and educational system. Subsequently, a decision was made not to develop a formal curriculum, but rather to develop national competence areas and goals for patient safety.

One of the primary purposes of developing national competence areas and goals is to define and frame patient safety as a distinct scientific field. This involves clarifying the scope of patient safety and establishing clear educational goals, competencies and standards that healthcare professionals must meet to ensure optimal patient care. By doing so, patient safety is recognised not just as a practice but as a critical area of scientific inquiry and professional development.

This work aims to describe the development of Swedish national competence goals in patient safety to establish patient safety as a distinct field summarised within a comprehensive framework of competency areas.

## Method

### Design

The development of national competence areas and goals was conducted from June 2023 to June 2024 using a modified Delphi methodology.

### Development of the national competence areas and goals

The development of national competence areas and goals was a part of the NBHW’s efforts to enhance knowledge and competence in patient safety. A modified Delphi process was employed to systematically collect insights from two multidisciplinary panels of experts.^9^ Originally developed in the 1950s, the Delphi method is a structured, iterative approach designed to build consensus among experts. The modified version of this method allows for flexibility in its design and implementation.^10^

#### Expert group and Delphi panels: recruitment and composition

The project was led by the NBHW and a Medical Officer from the NBHW served as the chair. A multiprofessional group of external experts (n=5) was commissioned to collaborate with officials at the NBHW (n=2) in designing the work and subsequently developing the national competence areas and goals. The external experts consisted of experts in patient safety, active at institutions conducting research and education in the field. This group was appointed to serve in the capacity of an expert group (table 1).

**Table 1:** Involved persons in the development of national competence areas and competence goals for patient safety

Expert group		
*Academic degree and professional background*	*Subject competency*	N
*National Board of Health and Welfare officials*		
Medical officer, MD	Patient safety	1
Medical officer, MD, PhD	Patient safety	1
*External experts*		
Professor, RN	Patient safety	1
Associate professor, psychologist	Patient safety	1
Associate professor, RN	Patient safety	1
Associate professor, MD	Patient safety	2
Total		7
Delphi panel I—subject content		
*Academic degree and professional background*	*Subject competency*	N
Associate professor, risk management engineering	Risk and safety science	1
PhD, engineering	Medical technology	1
PhD, MD	Healthcare hygiene	1
Associate professor, RN	Improvement and implementation science	1
Professor, engineering	Quality management	1
Professor, RN	Prehospital care	1
PhD, MD	Risk and safety science	1
Associate professor, MD	Artificial intelligence	1
PhD, RN	Caring sciences	1
PhD, psychologist	Human factors	1
Total		10
Delphi panel II—usability/target audience		
*Job position*	*Sector*	N
Chief nurse officer	The Swedish National Patient Insurance Company	1
Chief nurse officer	Municipality healthcare	1
Teacher/lecturer	RN education	3*
Teacher	Assistant nurse education	1*
Teacher/lecturer	Medical education	2
Chief medical officer	The Swedish National Patient Insurance Company	1
Professor (retired)	Research and teaching	1
Chief medical officer (retired)	Healthcare organisations	1
Programme or medical officer	National Board of Health and Welfare	3
Total		13

* One of the panellists was involved in both RN education and assistant nurse education.

MD, medical doctor; RN, registered nurse.

In addition, two multidisciplinary Delhi panels were established to support the project (table 1). The expert group identified potential panellists through established networks. They were purposefully selected to ensure representation from key stakeholder groups and to capture both practical and theoretical expertise. An invitation, including introductory information, was sent to 28 potential panellists, of whom 23 accepted to participate. No compensation was provided for their time. The first panel (n=10), consisting of experts with specific expertise in various competence areas, was tasked with providing subject-matter expertise related to these. The second panel (n=13) was assigned to provide feedback from the perspectives of different target user groups. These included representatives from institutions that provide education for healthcare professionals or individuals responsible for staff education in healthcare organisations.

#### The development process

The project was carried out in an iterative process, involving a series of physical (n=11) and digital meetings (n=4), as well as individual reading and work with texts between meetings. Additionally, two rounds of non-anonymous questionnaires were conducted, one for each Delphi panel.

First, a literature search was conducted to collect and assess documents containing learning objectives, patient safety competencies and curricula for patient safety from other countries and international organisations. This step was mainly for background oversight and inspiration.

The expert group then developed a first draft of competence areas, key concepts and competence goals in patient safety, based on the experts’ knowledge in the field, combined with insights from the literature search.

The draft and a questionnaire were sent via email to the first Delphi panel. The questionnaire consisted of a request for general comments, and four short essay questions (online supplemental table 1). Discussions were held, and oral feedback was given in a 2-hour digital meeting between the expert group and the panellists. Feedback was also provided from all panellists via written comments to the draft and answers to the questionnaire. No reminders were needed.

The feedback was compiled and analysed by the expert group, resulting in a revised version of the initial draft that was unanimously agreed on by all expert members. A written summary of the results was given to the first Delphi panel.

This revised version of the competence areas and goals was sent together with a questionnaire to the second Delphi panel. The questionnaire consisted of a request for general comments and seven short essay questions regarding the applicability and utility in patient safety education (online supplemental table 1). Feedback was provided from all panellists as written comments to the draft and answers to the questionnaire. No reminders were needed. The final version of the document was provided to the second panel.

Further revisions and finalisation of the competence areas, key concepts and competence goals were made after receiving feedback. None of these were removed, but the terminology and the description for some were revised, and the distinctions between certain competence areas were clarified. Consensus was reached within the expert group through both oral and written discussions. This process was carried out alongside ongoing alignment with national and international stakeholders and groups, as part of the NBHW’s coordinating role in implementing the national action plan for increased patient safety.

## Results

In total, 15 competence areas were created. Each competence area is described and summarised in the section below. An overview of these and the related key components (n=39) along with competence goals (n=113) is presented in online supplemental table 2. The full description of the competence areas can be found in online supplemental file 1.

### Competence area 1: patient safety – definitions, concepts and perspectives

This competence area focuses on the terminology and concepts central to patient safety, emphasising the need for a shared language to support healthcare practice, management and research. It examines the relationship between patient safety and other key aspects of Swedish healthcare, including person-centred and integrated care, quality and various safety domains (eg, work environment, radiation and information security). Additionally, it emphasises the importance of incorporating the perspectives of patients and their families, recognising that patient safety must be both systemically ensured and personally experienced by those receiving care.

### Competence area 2: responsibilities, obligations and roles in patient safety

This competence area addresses legal and organisational responsibilities for patient safety within healthcare. It encompasses knowledge of relevant laws and regulations, as well as their application to patient safety work at all levels, from local to international. It covers the responsibilities of individual practitioners, as well as those of governing bodies and healthcare providers. It focuses on reporting and incident investigation, risk management and promoting learning and development to enhance patient safety.

### Competence area 3: systems understanding, theories and frameworks

This competence area explores theories and frameworks for understanding healthcare as a complex adaptive system. It emphasises a systems perspective on patient safety, recognising that safety results from interactions between multiple actors and components, not just individual actions. The area highlights how variability and adaptability in healthcare affect both risks and opportunities for patient safety. It also covers the characteristics of organisations with strong safety awareness and effective risk management, aiming to support a holistic and dynamic approach to achieving and maintaining patient safety.

### Competence area 4: patients and their relatives as co-creators

This competence area focuses on engaging patients and their relatives in care, treatment and patient safety work at all levels of healthcare. It emphasises the patient as a co-creator, involved based on their conditions and preferences. It includes understanding how patient experiences and resources influence care and outlines the legal rights to participation in decisions that affect the design and delivery of care. It also covers how healthcare can support meaningful involvement in developing care processes and improving patient safety practices.

### Competence area 5: human factors

This competence area addresses how the physical, digital, organisational and social work environment impacts employees’ ability to ensure patient safety. It addresses how stress, fatigue and inadequate recovery affect cognitive function and decision-making. It emphasises integrating work environment and patient safety efforts, highlighting the importance of staff well-being for performance, recruitment and retention. The area includes concepts from Human Factors and Ergonomics, focusing on how the work environment influences health, cognition and safety outcomes.

### Competence area 6: teamwork and communication

This competence area emphasises the importance of teamwork and effective communication in enhancing patient safety. It includes team collaboration within and across care processes, involving both clinical and leadership levels. Effective communication with patients and their relatives fosters safety and participation. It emphasises how regular training in teamwork, including routine and unexpected situations, is essential. The area addresses organisational conditions, shared responsibilities, risk identification, joint decision-making and reflective learning. It also includes managing goal or value conflicts based on diverse perspectives from team members, patients and their relatives to ensure safe and coordinated care.

### Competence area 7: organisational culture and patient safety

This competence area focuses on how organisational culture influences patient safety and how it can be shaped to support it. Culture includes shared values, behaviours and assumptions that impact safety-related decisions, structures and practices. It can both promote and hinder patient safety. The area covers how to develop a safety-promoting culture, its link to safety climate and how to evaluate cultural strengths and weaknesses from a patient safety perspective using various methods. The competence area also involves evaluating organisational culture from a patient safety perspective, including the strengths and weaknesses, with different approaches.

### Competence area 8: risks and risk awareness

This competence area explores how risks arise, evolve and are managed in healthcare as a complex sociotechnical system. It addresses the interaction between people, technology and organisation, and how risks vary over time—some immediate, others long-term or unexpected. The area encompasses building risk awareness, maintaining safety at all levels and employing proactive methods to identify, analyse and manage risks, particularly during care transitions, organisational changes or resource shortages.

### Competence area 9: identify, investigate and learn from what has happened

This competence area is about identifying, reporting, investigating and learning from events that may impact patient safety, including preventable harm and near misses. It emphasises the legal framework for incident management and the importance of learning from both failures and successes. Feedback from analyses supports continuous improvement. The area also highlights the value of patients’ and their relatives’ experiences, viewpoints and concerns as key sources for learning and developing safer healthcare practices.

### Competence area 10: monitor and evaluate patient safety

This competence area focuses on monitoring and evaluating healthcare organisations from a patient safety perspective. It includes assessing safety presence, harm prevention and both proactive and reactive approaches. Follow-up can be based on care processes, patient experiences, operations or resource use. Collected data should guide systematic patient safety efforts by revealing risks, the effects of organisational changes and opportunities for improvement. The area also covers data sources, collection methods and their strengths and weaknesses.

### Competence area 11: safe processes and work practices

This competence area is about designing organisations, processes and work practices to enhance patient safety in healthcare settings of varying complexity. It includes integrating safety into the development and implementation of new practices through quality improvement, education and simulation. It also covers ‘de-implementation’ of outdated or risky processes, work practices and methods. Emphasis is also placed on ensuring secure information transfer and continuity of care, especially during transitions between units or organisations, to maintain patient safety throughout the care process.

### Competence area 12: technology and patient safety

This competence area emphasises ensuring patient safety throughout the entire life cycle of medical devices—from development and acquisition to use and phase-out. It addresses risks arising from the interaction between technology, humans and organisations, emphasising the importance of identifying and managing these risks. The user’s role is central, with a focus on user experience and workflow in design and implementation. It also encompasses legal regulations, standards and systematic methods for evaluating and monitoring patient safety related to the use of medical devices.

### Competence area 13: to lead and manage safe care

This competence area concerns how patient safety is led and organised in healthcare. It highlights the roles of leaders, managers and medical decision-makers in integrating patient safety into processes, decisions and changes. Leadership is recognised as crucial in building trust, facilitating risk management and supporting staff following adverse events. It also includes developing integrated management systems that combine patient safety, quality and work environment efforts, and using these systems to adapt goals and practices when demands exceed available resources.

### Competence area 14: emergency preparedness and patient safety

This competence area addresses how patient safety is impacted during crises, disasters and other extraordinary events. It covers how organisations shift from normal operations to crisis management and what that means for safety efforts. It includes principles for prioritisation, adapting healthcare goals through medical policy and ensuring emergency preparedness. Key elements are contingency planning, vulnerability analyses, maintaining competencies and using backup routines, crisis plans, inventory systems and disaster drills to support safe care under extraordinary conditions.

### Competence area 15: risk areas, areas of preventable patient harm and specific situations

This competence area addresses known risks and types of preventable patient harm. For several known risk areas, there is a reason to highlight specific competence needs. This area emphasises specific methods and practices to prevent, monitor and evaluate these risks. It highlights situations that require special competence, including challenging patient interactions influenced by factors such as age, language, health literacy or mental state. The area also encompasses patients who are unwilling or unable to participate in care. It acknowledges that risks and safety needs vary across different healthcare settings, necessitating tailored approaches for various organisations and contexts.

## Discussion

The work presented here aimed at developing competence goals for patient safety. The competence goals are organised in a framework of 15 competence areas. The competency areas in patient safety within the framework emphasise key elements, including systems thinking, teamwork, communication, human factors and organisational culture. This broad approach aligns with international trends that focus on integrating multiple concepts into patient safety education.^5^ We propose that this framework can be viewed as a description of patient safety as a scientific discipline, encompassing a wide range of educational goals, competencies and standards.^3^ This recognition is essential for fostering a shared understanding among healthcare professionals and stakeholders.^2^

A consistent perspective on patient safety, such as in the framework for competence goals presented here, supports the alignment of efforts across different levels of care, ensuring that patient safety is not only a practice but also a subject for continuous development and inquiry.^11^ The establishment of shared terminology and concepts facilitates collaboration and communication, which are critical for improving safety outcomes across healthcare systems.^1^

The 15 competence areas and the competence goals within them overlap in some aspects, and the division between them is not always clear-cut; this aligns with patterns observed in other frameworks for patient safety.^4 5^ It reflects the notion that healthcare has been described as a complex adaptive system,^12^ where organisations, practices and outcomes cannot be fully explained through linear thinking. Hence, any framework for patient safety knowledge must, by necessity, accommodate complexity and interconnections, rather than adhere to a strictly linear and compartmentalised structure.

The development of the competency goals in patient safety highlights the importance of reaching beyond traditional educational settings to include also healthcare professionals and leaders at all levels.^7^ By targeting not only students but also active practitioners and policymakers, the framework ensures that patient safety competence permeates all layers of healthcare. This model thus serves as a foundation for enhancing competence through the development of structured learning objectives tailored to diverse audiences.

Patient safety should be viewed as an ongoing process rather than a static goal. This requires sustained commitment to education, competence development and cultural change within organisations.^3^ Hence, patient safety education must be dynamic, incorporating advancements in safety science and addressing emerging challenges in healthcare delivery. The framework supports systematic efforts at all levels of healthcare to prevent harm and improve practices. By establishing foundational competency areas, the framework provides a basis for iterative development, ensuring its relevance over time. The framework emphasises the importance of locally developed learning objectives and progression that reflect diverse healthcare contexts, while remaining adaptable to new research and knowledge.^8 13^ This approach aligns with the recommendations from the WHO, which advocate for continuous updates to curricula based on evolving evidence.^4^

Given the variability in patient safety practices, tailored approaches are essential.^1^ While the framework offers a valuable starting point, its continued relevance depends on sustained efforts to integrate emerging research and adapt to evolving healthcare systems.

The broad scope of the framework may present challenges in its practical application. Ensuring that educational institutions and healthcare organisations have the competence to implement these goals in education requires significant investment in education and training infrastructure.^7^ Furthermore, there is a risk that local adaptations may dilute the consistency of patient safety knowledge if not carefully managed.^5^ While local adaptations allow for flexibility in addressing specific needs, they also pose risks if they are not aligned with established standards. Competence development must be prioritised to ensure that educators and healthcare leaders have the skills to build capacity effectively. Nonetheless, this challenge also presents an opportunity to innovate and refine patient safety education and practices through localised initiatives. Addressing these limitations necessitates coordinated governance and clear guidelines to support implementation.^8^

### Strengths and limitations

There is a lack of an established agreement on methods for developing competency frameworks for healthcare professionals.^14^ The iterative process used to develop the national competence goals demonstrates the value of collaboration among experts, stakeholders and panellists. One strength is the response rate since all panellists contributed with feedback, bringing broad and diverse subject expertise from various sectors, which offered a wide range of perspectives relevant to the framework’s development. We believe that the very high response and completion rate is based on the fact that all panellists saw the work with the national competence areas and goals as something very important for improvement of patient safety in Sweden. Heterogeneous panels may outperform homogeneous ones, making it important to include representation from relevant stakeholders,^15^ which we ensured in the present project.

A limitation is that we did not follow a strict Delphi protocol. Consistent with other studies employing a modified Delphi method, this project adapted the original approach, particularly regarding the definition of consensus and the handling of anonymity.^15^ Formal voting was not used because the questionnaires included essay questions. Instead, consensus was reached within the expert group, which, although made up of subject matter experts, may be considered a limitation. Furthermore, feedback was not anonymous, although written responses were not shared among panellists. However, we believe our approach ensures that the framework is both comprehensive and practical, addressing real-world challenges in patient safety education and implementation. Another limitation is that patients and/or the public were not involved in the project. However, the NBHW has a Patient and Carer Representatives Reference Group with which it maintains regular contact and is therefore aware of the viewpoints and perspectives on patient safety previously raised in this forum. These have been taken into account in the development process, although the reference group was not directly involved in the project and was instead kept informed of its progress.

## Conclusions

The development of national competence areas and goals represents a significant step toward establishing patient safety as a distinct scientific field, while addressing practical challenges in its implementation. By fostering collaboration across stakeholders and emphasising continuous improvement, this framework lays the groundwork for advancing patient safety education and practices.

The framework is designed to describe competence areas and goals. For implementation in education in different settings, curricula and learning objectives must be developed. Further studies focusing on its development, implementation and effect on patient safety practices and outcomes are necessary.

## Supplementary material

10.1136/bmjoq-2025-003887 online supplemental file 1

10.1136/bmjoq-2025-003887 online supplemental table 1

## Data Availability

All data relevant to the study are included in the article or uploaded as supplementary information.
