# Supplementary material for: Development of national competence areas and competence goals for patient safety using a modified Delphi method
Source: BMJ Open Qual. 2026 Mar 31;15(1):e003887. doi: 10.1136/bmjoq-2025-003887 (PMC13052552; doi:10.1136/bmjoq-2025-003887)
Supplement: online supplemental file 1 [file bmjoq-15-1-s001.docx]

# The 15 competence areas, full description

In total, 15 competence areas were created with related key components (n=39) and competence goals (n=113).

## Competence area 1. Patient safety: definitions, concepts, and perspectives

This competence area addresses the terms and concepts used in the field of patient safety, and how they are defined and applied. It is essential to establish a common terminology:

- In the work related to patient safety in healthcare facilities
- In management to enhance patient safety
- When reading, writing, and researching within the field of patient safety.

The area covers how patient safety relates to other perspectives within the Swedish healthcare, including hospital care and primary care, such as person-centred and integrated care, as well as quality. It also addresses how patient safety intersects with other safety efforts in healthcare, including work environment, operational and functional safety, radiation safety, and information security.

Furthermore, this competence area encompasses patient safety from the perspective of patients and their relatives, which is a crucial complement to the healthcare system’s viewpoint. For patients, the sense of safety is vital. Therefore, patient safety is not only about ensuring safety from the healthcare system’s viewpoint; it must also be perceived as safe by patients and their relatives.

## Competence area 2. Responsibilities, obligations, and roles in patient safety

This competence area focuses on the responsibilities and obligations related to patient safety for employees and officials at various levels within the healthcare system.

The area of competence is based on legal regulation and the knowledge of how it is applied in work related to patient safety. Legal regulation is a collective term for various types of regulations, including laws, ordinances, regulations, and general guidelines.

The area also includes knowledge about how patient safety work is organised and conducted at local, regional, national, and international levels.

The competence area covers the responsibilities of individual practitioners, as well as those of governing bodies and healthcare providers. Topics addressed within this area may include:

- Ensuring high patient safety and compliance with reporting obligations
- Organising systematic patient safety efforts, such as incident reporting
- Investigating, assessing, and managing events and risks
- Establishing structures for learning and professional development in patient safety
- Reporting and notifying supervisory authorities.

## Competence area 3. Systems understanding, theories, and frameworks

This competence area focuses on theories and frameworks for describing and understanding healthcare from a system perspective. A system perspective may contribute to understanding, describing, simplifying, and gaining an overview of patient safety within healthcare.

The perception of patient safety has evolved over time. Healthcare has shifted from an individual-centric view of right or wrong actions to an understanding that other underlying factors influence patient safety. It is the sum of various components within the healthcare system.

System understanding is based on describing healthcare as a complex adaptive system. This means that different parts and levels of healthcare interact and influence each other. These interactions can occur unpredictably, necessitating continuous adaptations. Different parts of the healthcare system carry their respective risks and challenges. Therefore, various approaches are needed to achieve and maintain patient safety.

This competence area also addresses how the healthcare system’s variability and adaptability impact opportunities and respective challenges related to patient safety. Additionally, it includes characteristics of organisations with a high awareness of safety and a strong ability to manage risks.

## Competence area 4. Patients and their relatives as co-creators

This competence area focuses on involving and engaging patients and their relatives in care and treatment, as well as in systematic patient safety work and the design of healthcare at all levels.

It concerns the participation of patients and their relatives as individuals, as a group, and/or as representatives of patient or relative organisations.

The patient as a co-creator means that the patient is involved at all levels of the healthcare system, based on their own wishes and conditions for participation. It is essential to have knowledge about how the patient’s narrative and resources are utilised in the management and design of care.

Patients’ opportunities to participate in and influence specific issues that directly affect the design of care are clearly outlined in Swedish legislation. This includes matters such as access, the provision of specific information to the patient, and the patient’s involvement in the design and provision of care. The patient’s relatives should also have the opportunity to participate in the design and delivery of care, if it is appropriate and if provisions of confidentiality or professional secrecy do not prevent this.

The competence area also encompasses how healthcare can foster conditions for patients and their relatives to participate in and contribute to systematic patient safety work. This can be achieved by involving patients and their relatives in the development of care processes and work practices.

## Competence area 5. Human factors

The area of competence concerns how the work environment affects employees' ability to work in a way that promotes patient safety. It includes the physical (including digital), organisational, and social work environment. For example, an imbalance between demands and resources can cause fatigue and stress. The same applies to the opportunity for recovery during and between work shifts. This, in turn, affects cognitive abilities, situational awareness, and the ability to make decisions. These are factors that are crucial for performing work in a manner that contributes to patient safety.

The competence area also involves integrating systematic work environment efforts with systematic patient safety work. The work environment, including how work is organised and how events are handled, has consequences for employees’ health and well-being. These factors are also important for recruiting and retaining competent staff. The area encompasses what is commonly referred to internationally as "Human Factors" and the field of Ergonomics, i.e., how the work environment affects employees' cognitive ability, as well as their physical and mental health.

## Competence area 6. Teamwork and communication

This competence area focuses on how teamwork and communication contribute to patient safety, as well as communication within and between teams. It also concerns how good communication with patients and their relatives can contribute to a sense of safety, comprehensibility, and participation, which in turn contributes to patient safety.

The team is one of the most common ways of organising work in healthcare. Teams may look different in different operations. They can be permanent or temporary, physical or virtual. They can be located near the patient or serve as a bridge between different parts of the patient’s care process. Management teams and teams at operational and political levels also affect patient safety.

A prerequisite for effective teamwork that promotes patient safety is regular training. This involves training in both everyday routine situations and the ability to handle and recover from disturbances and unexpected situations. Deficiencies in teamwork and communication are common contributing causes of preventable patient harm. Therefore, the competence area addresses organisational conditions for teamwork, as well as how team members contribute their respective professional competencies to the work.

Common work practices in team situations are also included. These work practices involve:

- Collaborating and communicating
- Establishing a sense of safety in the team
- Formulating goals and maintaining a common understanding of the task
- Identifying risks
- Making decisions based on common priorities
- Reflecting and learning together after both every day and escalating situations.

The competence area also deals with the ability to recognise and manage goals and value conflicts based on the team’s, the patient’s, and the relatives’ different perspectives on the situation.

## Competence area 7. Organisational culture and patient safety

This competence area concerns how organisational culture affects patient safety and how it can be developed to promote patient safety.

Organisational culture can be described as the fundamental values, assumptions, and behaviours that are shared by the people within an organisation. The culture of an organisation influences how we perceive issues that are significant for developing and maintaining patient safety. This includes competence at both the individual and group levels, as well as structures, processes, technology, and behaviours. The culture can both promote and hinder patient safety. Therefore, the competence area also deals with:

- How an organisational culture is developed
- What is meant by an organisational culture that promotes patient safety
- How it can contribute to promoting or hindering the development of patient safety
- The concept’s relation to the concept of safety climate.

## Competence area 8. Risks and risk awareness

This competence area addresses how risks emerge, evolve, and transform over time in healthcare. Healthcare can be described and understood from a systems perspective as a complex sociotechnical system. This means that different parts and levels of healthcare interact and influence each other, and that there is an interplay between humans, technology, and organisation throughout all parts.

Risks develop over different time perspectives. Some risks occur in the present moment, in daily work, while others emerge over a longer period. Some risks are anticipated, while others are not. Therefore, the area also addresses risk awareness and how patient safety is continually created and maintained at all levels of the healthcare system.

The competence area also involves identifying, analysing, and managing risks, as well as anticipating and handling variations that occur in daily work. Proactive approaches within different parts of healthcare and for various risk situations, such as care transitions, during organisational changes, or when there is a shortage of hospital beds, are also included.

## Competence area 9. Identify, investigate, and learn from what has happened

This competence area focuses on identifying, reporting, investigating, and learning from events in healthcare that may impact patient safety. It encompasses events that have resulted in or could have resulted in preventable patient harm or other incidents. Incident management and investigations are legally regulated areas. Central to the learning process are the factors that contribute to maintaining or strengthening patient safety.

Feedback in the form of analysis of results and conclusions after events creates conditions for continuous learning. They also provide a basis for systematic improvement work at all levels of healthcare.

The area also involves utilising the experiences, viewpoints, and complaints of patients and their relatives. They are a source of learning and development in patient safety work.

## Competence area 10. Monitor and evaluate patient safety

This competence area encompasses various aspects, perspectives, and measures for monitoring and evaluating healthcare organisations from a patient safety perspective at all levels. Examples of aspects include the presence of safety, the absence of preventable patient harm, and proactive and reactive approaches. Different perspectives on follow-up could, for example, include operational, care, patient, and resource perspectives.

Measurements and analysis of collected data need to be the basis for managing and organising systematic patient safety work. Data, for example, can show how organisational conditions, changes within organisations, and behaviours affect patient safety risks. Data can also indicate opportunities for improvement work. Therefore, the competence area also includes data sources and methods for data collection, as well as their strengths and weaknesses.

## Competence area 11. Safe processes and work practices

This competence area focuses on how organisations, processes, and work practices can be designed to enhance patient safety across various operations with varying degrees of complexity. The area includes how patient safety aspects can be highlighted and managed when new processes and work practices are developed and implemented. This can be achieved, for example, through systematic quality improvement work, as well as clinical education, training, and simulation. The competence area also encompasses “de-implementation,” which involves phasing out processes, work practices, and methods that no longer create value or pose unacceptable risks.

This competence area also emphasises processes and work practices for secure information transfer and continuity, which are particularly relevant when a patient is transferred between care units or healthcare organisations, or when multiple care units are involved in the patient's care.

## Competence area 12. Technology and patient safety

This competence area is about considering patient safety at all levels when medical devices are developed, acquired, introduced, used, and phased out in healthcare. In the interplay between technology, humans, and organisations, risks can arise. They need to be identified and managed. This applies to all parts of a medical device’s life cycle.

The competence area also involves viewing the user as an active participant throughout the entire life cycle of medical devices. This can be done, for example, by considering the user’s experience and workflow throughout the whole design and implementation process. It can also involve using a user-oriented method when introducing new technology.

The competence area also includes legal regulations and standards in the field of medical technology, as well as methods for systematically evaluating and monitoring patient safety related to the use of medical devices.

## Competence area 13. To lead and manage safe care

This competence area focuses on how patient safety work is managed and organised in healthcare. It addresses how decision-makers, leaders, managers, and individuals with medical management responsibilities can collaborate to prioritise and integrate patient safety into work processes, decisions, and organisational changes.

It also involves the roles of formal and informal leadership as culture-creating forces. This includes work practices to:

- Create trust and a sense of safety in the organisation
- Provide conditions for risks to be noticed, communicated, and managed
- Support employees who have been involved in events that have or could have led to preventable patient harm.

The competence area also includes how an integrated management system, where management systems for patient safety are integrated with management systems for quality and work environment, can be created and translated into practical patient safety work. It also addresses how a management system can help identify the need to change work practices, adapt goals, and reprioritise when demands exceed resources, a situation that can arise both in normal conditions and during crises of varying magnitudes.

## Competence area 14. Emergency preparedness and patient safety

This competence area addresses how patient safety efforts are impacted during extraordinary events, crises, and disasters. It includes how an organisation can identify the need to switch work practices from normal operations to management of crises of different magnitudes, what this entails, and what it means for patient safety work.

The area also concerns principles for prioritisation during crises of different magnitudes and how healthcare goals can be adapted through medical policy decisions. The area also includes patient safety aspects of emergency preparedness work. This can involve:

- Contingency planning
- Vulnerability analyses
- How competencies and work practices in crisis or disaster situations are maintained through backup routines, crisis plans, crisis organisation, inventory management, and disaster exercises.

## Competence area 15. Risk areas, areas of preventable patient harm, and specific situations

The competence area deals with known risks and preventable patient harm areas. For several known risk areas, there is a reason to highlight specific competence needs. There is also knowledge about different types of preventable patient harm and how they can be prevented. For many of these risk areas and types of preventable patient harm, specific methods and work practices are available to mitigate them. It is about methods and work practices to prevent risks and preventable patient harm, and to follow and evaluate care from a patient safety perspective.

The competence area also addresses care situations that can present challenges. The way patients are treated is fundamental for fostering a trusting and mutually respectful relationship between healthcare providers and patients. It is central for achieving participation and a sense of safety. Factors such as age, gender, language, health literacy, socioeconomic status, functional level, and religious beliefs can affect the meeting between the patient and healthcare workers. Inadequate attitude towards patients can result in poor communication and information exchange, which can subsequently lead to patient safety risks.

There are also care situations that can be challenging because the patient has difficulty to, or does not want to, participate in their care and treatment due to illness, health condition, or other conditions. It can also include patients who are a risk to themselves or others. Examples are patients with acute confusion, dementia, psychosis, substance influence, among others.

Risks and preventable patient harm manifest differently across various parts of the healthcare system. Therefore, the competence needs differ across various organisations and types of healthcare. The examples are intended to illustrate which risk areas, areas of preventable patient harm and specific care situations may be relevant.

The examples do not claim to be complete.
