# Supplementary material for: Development of national competence areas and competence goals for patient safety using a modified Delphi method
Source: BMJ Open Qual. 2026 Mar 31;15(1):e003887. doi: 10.1136/bmjoq-2025-003887 (PMC13052552; doi:10.1136/bmjoq-2025-003887)
Supplement: online supplemental table 1 [file bmjoq-15-1-s002.docx]

# Supplemental tables

| **Supplemental table 1** Essay questions in questionnaires in the two Delphi panels |
| --- |
| **Delphi panel 1** |
| 1. What are your general comments on the framework of competence areas and goals? 2. The competence areas and goals aim to describe patient safety as a knowledge domain as a whole. Do you think any content is missing? 3. Are your competence area(s) (defined by you as you see fit) described adequately, and do the goals capture the most essential aspects within each area? 4. Do you have comments or suggestions for improving the wording of the descriptive texts regarding competence areas or goals relevant to you? 5. Additional question for those concerned: Would the competence goals be supportive and useful for you in designing education on patient safety? |
| **Delphi panel 2** |
| 1. What are your general comments to the framework of competence areas and goals? 2. From your perspective, how can the competence goals serve as support when developing education in patient safety? 3. Is there anything that should be clarified, or that is described in a way that is too theoretical or complicated? 4. Is the structure with A, B, and C goals sufficiently clear in terms of how it works and is intended to be used when developing education for different professional groups? 5. Do you see a need for additional examples or illustrations to make it easier for the reader? 6. In your view, what is needed going forward to ensure that the competence goals are used and achieve their intended effects? 7. Do you see any obstacles or opportunities that the National Board of Health and Welfare should take into account in the continued work? 8. Do you have any other comments or feedback on the draft national competence goals? |

| **Supplemental table 2** Overview of the 15 competence areas and related key components with competence goals | |
| --- | --- |
| **Competence areas** | **Key components with competence goals** |
| 1. Patient safety: definitions, concepts, and perspectives | ***Terms and concepts***   - Demonstrate knowledge of how patient safety terms and concepts are defined and used. - Be able to use patient safety terms and concepts.   ***Patient safety in relation to other concepts and dimensions within healthcare***   - Demonstrate knowledge of how patient safety concepts relate to other concepts of quality in healthcare. - Demonstrate knowledge of how patient safety is related to other safety efforts within healthcare.   ***Patient safety from the patient’s and their relatives’ perspective***   - Demonstrate knowledge of how the meaning of patient safety from the perspective of patients and their relatives complements the healthcare perspective. |
| 2. Responsibilities, obligations, and roles in patient safety | ***The legal regulation of patient safety***   - Demonstrate knowledge of the legal regulations that define patient safety and how it is applied in work related to patient safety - Demonstrate knowledge of the responsibilities and duties of different actors, including individual practitioners, governing bodies, healthcare providing organisations, and management at different levels in the healthcare system, regarding patient safety in accordance with legal regulations in the field. - Demonstrate knowledge of methods and work practices related to the responsibilities and duties of different actors, including individual practitioners, governing bodies, healthcare providing organisations, and management at different levels in the healthcare system, regarding patient safety in accordance with legal regulations in the field.   ***Organisation of work with patient safety***   - Demonstrate knowledge of the organisation of local, regional and national patient safety work. |
| 3. System understanding, theories, and frameworks | ***Evolution of the concepts of safety over time***   - Demonstrate knowledge of how the understanding of safety, with regard to the individual and the system, influences the patient safety work. - Demonstrate knowledge of how the understanding of patient safety, regarding the individual and the system, has evolved.   ***System understanding***   - Demonstrate knowledge of how different theoretical frameworks and models of systems can contribute to patient safety work.   ***Safety in complex systems***   - Demonstrate knowledge of how the healthcare system can be understood as a complex adaptive system. - Demonstrate knowledge of how variability within complex adaptive systems impacts possibilities and limitations in patient safety work. |
| 4. Patients and their relatives as co-creators | ***Involve patients and their relatives in care and treatment***   - Demonstrate knowledge of how involving patients and their relatives in issues that directly impact care and treatment promotes patient safety. - Demonstrate knowledge of methods and work practices that create conditions for patients´ and their relatives´ involvement in decision-making and the provision of care and treatment, when possible. - Be able to create conditions for the involvement of patients and their relatives in decision-making and the provision of care and treatment, when possible.   ***Involve patients and their relatives in the systematic patient safety work.***   - Demonstrate knowledge of how the involvement of patients and their relatives promotes systematic patient safety work. - Demonstrate knowledge about methods and work practices for involving patients and their relatives in the systematic patient safety work. - Be able to involve patients and their relatives in the systematic patient safety work.   ***Involve patients and their relatives in the design of care at all levels.***   - Demonstrate knowledge of the involvement of patients and their relatives in the design of care at all levels to promote patient safety. - Demonstrate knowledge of methods and work practices for involving patients and their relatives in the design of care at all levels. - Be able to involve patients and their relatives in the design of care at all levels. |
| 5. Human factors | ***Physical, organisational, and social work environment***   - Demonstrate knowledge of how the design of the physical work environment affects employees’ conditions and ability to work in a way that promotes patient safety. - Demonstrate knowledge of methods and work practices that contribute to the physical, organisational and social work environment, strengthening the employees' ability to work in a way that promotes patient safety. - Be able to apply methods and work practices that contribute to strengthening the physical, organisational and social work environment, thereby enhancing the employees' ability to work in a way that promotes patient safety.   ***How situational awareness and decision-making are affected by stress and fatigue***   - Demonstrate knowledge of how stress and fatigue affect a person´s situational awareness, decision-making, as well as the ability to work in a way that promotes patient safety. - Demonstrate knowledge of methods and work practices to reduce and manage stress and fatigue, promoting abilities such as situational awareness and decision-making. - Be able to apply methods and work practices to reduce and manage stress and fatigue to promote abilities such as situational awareness and decision-making.   ***Well-being and work capacity after involvement in events that have or could have led to preventable harm***   - Demonstrate knowledge of how well-being and work ability are affected by being involved in events that have resulted in or could have resulted in preventable patient harm. |
| 6. Teamwork and communication | ***Teams in healthcare***   - Demonstrate knowledge of the different types of teams within healthcare, the contexts in which teams operate, the characteristics of effective teamwork, and how teamwork impacts patient safety - Be able to apply methods and work practices for teamwork that promote patient safety. - Demonstrate knowledge of how different professions and roles in healthcare contribute complementary perspectives and competencies. - Be able to identify, communicate and manage goal- and value conflicts based on different perspectives in the team.   ***Communication and patient safety***   - Demonstrate knowledge of how communication can contribute to a sense of safety, comprehensibility and involvement within and between teams, as well as in interactions with patients and their relatives, and during transitions in care. - Demonstrate knowledge of methods and work practices for communication within and between teams, with patients and their relatives, as well as during transitions in care, that promote patient safety. - Be able to apply methods and work practices for communication within and between teams, with patients and their relatives, as well as during transitions in care that promote patient safety. |
| 7. Organisational culture and patient safety | ***The potential of organisational culture to promote or hinder patient safety***   - Demonstrate knowledge of what characterises an organisational culture that supports patient safety at different levels in healthcare. - Demonstrate knowledge of methods and work practices at different levels in healthcare to create an organisational culture that promotes patient safety. - Be able to apply methods and work practices at different levels in healthcare to create an organisational culture that promotes patient safety.   ***To evaluate how organisational culture affects patient safety***   - Demonstrate knowledge of measurable aspects of organisational culture. - Demonstrate knowledge of methods and work practices for evaluating the organisational culture in relation to patient safety. - Be able to evaluate the organisational culture regarding patient safety, considering the different possibilities and limitations of methods. |
| 8. Risks and risk awareness | ***Risks in complex sociotechnical systems***   - Demonstrate knowledge that patient safety risks vary across different areas of healthcare, and that work practices need to be adapted both to the nature of the risks and to the conditions of the organisation. - Demonstrate knowledge of how risks emerge, develop and change over time in complex sociotechnical systems.   ***Risk awareness***   - Demonstrate knowledge of what impact risk awareness and a proactive approach in care and treatment, as well as in risk situations, have at different levels and within various types of healthcare. - Demonstrate knowledge of methods and work practices for identifying, analysing and managing safety risks both in the short and long term, as well as at different levels and within various types of healthcare. - Be able to identify, analyse and manage safety risks both in the short and long term, as well as at different levels and within various types of healthcare. |
| 9. Identify, investigate, and learn from what has happened | ***Identify and report***   - Demonstrate knowledge of how identifying and reporting events impact patient safety. - Demonstrate knowledge of methods for identifying and reporting events that impact patient safety. - Be able to identify and report events that impact patient safety.   ***Investigate events***   - Demonstrate knowledge about how investigations of events contribute to improved patient safety. - Demonstrate knowledge of methods for investigating events that impact patient safety and quality of care. - Be able to apply methods to investigate events that impact patient safety.   ***Utilise patients’ and their relatives’ experiences, views, and complaints***   - Demonstrate knowledge of how utilising patients' and their relatives' experiences, views, and complaints contributes to improved patient safety. - Demonstrate knowledge of methods and work practices for utilising patients' and their relatives' experiences, views, and complaints (to improve patient safety). - Be able to apply methods for utilising patients' and their relatives' experiences, views, and complaints (to improve patient safety).   ***Feedback and learning for improved patient safety***   - Demonstrate knowledge of how feedback and learning can contribute to improved patient safety. - Demonstrate knowledge of methods and work practices for providing feedback and learning from events at all levels within healthcare - Be able to apply methods and work practices for providing feedback and learning from events at all levels within the healthcare system. |
| 10. Monitor and evaluate patient safety | ***Monitor and evaluate patient safety.***   - Demonstrate knowledge of how follow-up and evaluation of healthcare from a patient safety perspective contribute to increased patient safety. - Demonstrate knowledge of different aspects, perspectives, and measures for monitoring and evaluating healthcare as a basis for systematic patient safety work. - Demonstrate knowledge of data sources, as well as methods for data collection and analysis, to monitor and evaluate healthcare from a patient safety perspective, their strengths and weaknesses, and the implications of variation. - Be able to apply methods for data collection and analysis to monitor and evaluate healthcare from a patient safety perspective. |
| 11. Safe processes and work practices | ***Designing organisations, processes, and work practices in complex systems***   - Demonstrate knowledge of how the design of organisations, processes, and work practices impacts patient safety in the implementation and outcomes of healthcare. - Demonstrate knowledge of methods and work practices for how processes and work practices in healthcare can be designed and adapted to function in different organisations and under changing conditions. - Be able to apply methods and work practices for how processes and work practices in healthcare can be designed and adapted to function in different organisations and under changing conditions.   ***Implementation and de-implementation of processes, work practices, and methods***   - Demonstrate knowledge of patient safety aspects when implementing new processes, work practices, and methods, as well as when de-implementing processes, work practices, and methods that do not create value or that involve too significant risks. - Demonstrate knowledge of methods and work practices for the implementation and de-implementation of processes, work practices, and methods. - Be able to apply methods and work practices for the implementation and de-implementation of processes, work practices, and methods.   ***Secure information transfer and continuity during care transitions within and between care providers***   - Demonstrate knowledge of how information security can both contribute to and hinder safety care. - Demonstrate knowledge of how systems for documentation and information transfer can be designed for safe care. - Demonstrate knowledge of how collaboration and information transfer within and between healthcare providers promote safe care transitions. - Demonstrate knowledge of methods and work practices that promote collaboration and information transfer to ensure safe care transitions within and between healthcare providers. - Be able to apply methods and work practices for collaboration and information transfer that promote safe care transitions within and between healthcare providers. |
| 12. Technology and patient safety | ***The interaction between humans and technology and the importance of a user perspective throughout the entire life cycle of a medical device***   - Demonstrate knowledge of the interaction between humans, medical technology, and organisation, and its significance for risks and patient safety. - Demonstrate knowledge of methods and work practices for identifying, analysing and managing risks based on the interaction between humans, medical technology, and organisation. - Be able to identify, analyse, and manage risks based on the interaction between humans, medical technology, and the organisation during the development, procurement, implementation, and/or use of medical devices.   ***Standards and regulations for medical devices***   - Demonstrate knowledge of standards and regulations regarding medical devices. - Be able to use methods and regulations to promote patient safety when procuring medical devices as well as when using medical devices in clinical care. |
| 13. To lead and manage safe care | ***Leadership for safe care***   - Demonstrate knowledge of how leadership influences and creates conditions for patient safety. - Demonstrate knowledge of methods and work practices for safety-oriented leadership. - Be able to apply methods and work practices for safety-oriented leadership, and lead and delegate tasks in a way that promotes patient safety.   ***Management systems for patient safety***   - Demonstrate knowledge of how systematic patient safety work is organised and managed within organisations that provide healthcare, including the legal regulations. - Demonstrate knowledge of what an integrated management system is, and the key roles and functions required for systematic patient safety work. - Demonstrate knowledge of methods and work practices for the integration of patient safety into a management system. - Be able to apply methods and work practices to integrate patient safety into a management system.   ***Management systems for various conditions***   - Demonstrate knowledge of how healthcare organisations may need to shift work practices and goals in situations where needs exceed resources, and how this impacts patient safety. - Demonstrate knowledge of methods and work practices to adapt work practices and goals to different operational situations with a focus on patient safety and how it is impacted. - Be able to apply methods and work practices to adapt work practices and goals to different operational situations.   ***Support for employees***   - Demonstrate knowledge of how employees' well-being and work ability are affected by being involved in events that have resulted in or could have resulted in preventable harm to a patient. - Demonstrate knowledge of methods and work practices for organising support interventions for employees involved in events that have resulted in or could have resulted in preventable patient harm. - Be able to apply methods and work practices for supporting interventions for employees involved in events that have resulted in or could have resulted in preventable patient harm can be organised. |
| 14. Emergency preparedness and patient safety | ***Patient safety in crisis or disasters***   - Demonstrate knowledge of various aspects of how patient safety and patient safety work are impacted in a crisis or disaster situation. - Demonstrate knowledge of prioritizations in a crisis or disaster situation and how healthcare can be adapted through medical policy decisions. - Demonstrate knowledge of how work practices in healthcare can be adapted to maintain patient safety. - Demonstrate knowledge of how competencies and work practices in crisis or disaster situations are maintained through backup routines, crisis plans, crisis organisations, inventory management, and disaster training. - Be able to apply methods that contribute to maintaining competencies and work practices for patient safety during a crisis or disaster.   ***Contingency planning and vulnerability analyses***   - Demonstrate knowledge of methods for contingency planning and vulnerability analyses. - Be able to apply methods for contingency planning and vulnerability analyses. |
| 15. Risk areas and preventable patient harm, and specific situations | ***Specific risk areas*** Some examples (not exhaustive) include diagnostics, digital healthcare, medication management, home healthcare, surgical procedures, and transitions of care.   - Demonstrate knowledge of legal regulations that define and govern specific risk areas. - Demonstrate knowledge of areas within healthcare where specific patient safety risks exist. - Demonstrate knowledge of methods and work practices to prevent risks and preventable patient harm within the specific risk area. - Be able to apply methods and work practices to prevent risks and preventable patient harm within the specific risk area.   ***Preventive work within specific areas of preventable patient harm*** Some examples (not exhaustive) include fall prevention, pressure ulcer prevention, prevention of bladder overdistension, prevention of healthcare-associated infections, and prevention of suicide as a healthcare-related harm.   - Demonstrate knowledge of types of preventable harm that can affect patients. - Demonstrate knowledge of methods and work practices to prevent risks and preventable patient harm, as well as to monitor and evaluate care from a patient safety perspective within specific areas of preventable harm. - Be able to apply methods and work practices to prevent risks and preventable patient harm within the specific areas of preventable harm.   ***Care situations with specific patient safety challenges*** Some examples (not exhaustive) include   - Situations where age, health literacy, socioeconomic status, language, religious beliefs, physical or intellectual functioning, and similar factors   may pose a risk for unjustified differences in care and treatment.  - Situations where the patient may pose a threat to themselves or others.  - Situations where protective and restraining measures may be necessary.  - Situations where healthcare staff are confronted with threats or violence.   - Demonstrate knowledge of care situations that may involve specific patient safety challenges and the associated legal regulations. - Demonstrate knowledge of methods and work practices that can be used in care situations with specific patient safety challenges. - Be able to apply methods and work practices that can be used in care situations with specific patient safety challenges. - Demonstrate knowledge of how attitude and trust-building can contribute to improved patient safety. - Demonstrate knowledge of methods and work practices that can contribute to building trust and enhancing the patient´s sense of safety in the care situation. - Be able to apply methods and work practices that can help build trust and strengthen the patient's sense of safety in the care situation. |
